# Genome analysis provides insight into hyper-virulence of *Streptococcus suis* LSM178, a human strain with a novel sequence type 1005

**DOI:** 10.1038/s41598-021-03370-0

**Published:** 2021-12-14

**Authors:** Yong Hu, Shiming Fu, Geng Zou, Anusak Kerdsin, Xiabing Chen, Xingxing Dong, Lin Teng, Jinquan Li

**Affiliations:** 1 Key Laboratory of Fermentation Engineering (Ministry of Education), Hubei Key Laboratory of Industrial Microbiology, National “111” Center for Cellular Regulation and Molecular Pharmaceutics, Hubei Research Center of Food Fermentation Engineering and Technology, Hubei University of Technology, Wuhan, 430068 Hubei People’s Republic of China; 2 Key Laboratory of Environment Correlative Dietology, College of Food Science and Technology, Huazhong Agricultural University, Wuhan, 430070 Hubei People’s Republic of China; 3 Faculty of Public Health, Kasetsart University Chalermphrakiat Sakon Nakhon Province Campus, Sakon Nakhon, 47000 Thailand; 4 Institute of Animal Husbandry and Veterinary Science, Wuhan Academy of Agricultural Sciences, Wuhan, 430208 Hubei People’s Republic of China

**Keywords:** Comparative genomics, Bacteria

## Abstract

*Streptococcus suis* has been well-recognized as a zoonotic pathogen worldwide, and the diversity and unpredictable adaptive potential of sporadic human strains represent a great risk to the public health. In this study, *S. suis* LSM178, isolated from a patient in contact with pigs and raw pork, was assessed as a hyper-virulent strain and interpreted for the virulence based on its genetic information. The strain was more invasive for Caco-2 cells than two other *S. suis* strains, SC19 and P1/7. Sequence analysis designated LSM178 with serotype 2 and a novel sequence type 1005. Phylogenetic analysis showed that LSM178 clustered with highly virulent strains including all human strains and epidemic strains. Compared with other strains, these *S. suis* have the most and the same virulent factors and a type I-89 K pathogenicity island. Further, groups of genes were identified to distinguish these highly virulent strains from other generally virulent strains, emphasizing the key roles of genes modeling transcription, cell barrier, replication, recombination and repair on virulence regulation. Additionally, LSM178 contains a novel prophage conducive potentially to pathogenicity.

## Introduction

*Streptococcus suis* (*S. suis*) is one of the most important swine pathogens leading to severe economic losses to the porcine industry worldwide. However, *S. suis* has emerged as a zoonotic agent, causing fever, septicemia, meningitis, arthritis and a variety of other symptoms in humans^1^. Since the first case of human *S. suis* infection reported in 1968^2^, it has spread in more than 30 countries and regions, particularly the southeast Asian countries where the pathogen represents a significant public health concern^3,4^. Two serious outbreaks have occurred in China (Sichuan in 2005 and Jiangsu in 1998)^5^ and four in Thailand (Phayao in 2007, Chiang Mai and Lamphoon in 2008, Phetchabul in 2010 and Uttaradit in 2019)^6^. A recent study reported that, upon testing of raw pork and edible pig organs collected from 88 sales locations in central Thailand, the positive rate of *S. suis* was as high as 85.23% and the positive rate of serotype 2 was 17.05%^7^. In addition, 70.4% of *S. suis* isolates of serotypes 2 and 14 from slaughtered pigs revealed sequence types and pulsotypes identical to human isolates in Thailand, suggesting transmission from pigs to humans^8^.

Typing of *S. suis* strains is epidemiologically important to control the infection. The most commonly used method, serotyping, is not only used for identification and diagnosis of clinical of *S. suis* isolates, but has also been suggested to be indicative of pathogenicity^6^. Of the 35 serotypes (types 1–34 and 1/2) originally identified according to the antigenicity of capsular polysaccharide (CPS), six *S. suis*-like strains (serotypes 20, 22, 26, 32, 33, and 34) have been taxonomically removed from the *S. suis* species based on phylogenetic and sequence analyses^9^. Additionally, new variants with serotype Chz and novel cps loci were recently investigated, although their relationship to virulence potential remains unclear^10–13^. The prevalence of *S. suis* serotypes in countries and regions is different. For instance, isolates associated with pig disease were predominantly identified as serotypes 2 and 9 in Europe^14^, and serotypes 2 and 3 in North America^15^. However, serotype 2 is considered to be the most toxic and prevalent serotype causing both pig and human infection worldwide^16,17^, although other serotypes such as serotype 9 and 14 are of increasing concern^18^.

Besides serotyping, genetic classification by multiple sequence locus typing (MLST)^19^ has become increasingly important because of its higher resolution for investigating strain evolution and delineating the relationship between subtype and pathogenicity. For instance, while serotype 2 ST1 strains present high zoonotic potential worldwide, ST7 from serotypes 2 and 14 is common in China^6,20^. And, in Thailand for human infections, ST104 are almost exclusively predominant in serotype 2 and the majority of serotype 14 isolates are ST105^6^. So far as to April 8, 2021, 2,808 STs have been recorded in the *S. suis* MLST database, showing that *S. suis* is constantly evolving. Though only a small number of STs have been found to be mainly responsible for human infections, the increasing diversity brings new risks and challenges, such as ST658 isolated in China. The strain originates from the ST1 complex and showed more mortality rates (90%) than the international reference virulent strain P1/7 (ST1) (70%) in a murine model^21,22^.

There is no doubt that the repertoire of *S. suis* virulence determinants play a role in human infection, since *S. suis* was suggested to be a cause of community-acquired disease^23^. It is difficult to make any clear distinction about the virulence factors belonging exclusively to pigs versus humans. In a recent study, no defined genomic differences between human strains and pig strains were suggested, although human disease isolates are limited to a single virulent population whose origin nevertheless coincided with the first intensification of pig production^24^. Another study found that virulent strains could not be identified only by the presence of proposed virulence factors, making the definition of virulence factors ambiguous^25^. Virulence factors play key roles in many aspects. One reason why serotype 2 strains show high zoonotic potential and virulence in humans is their better adherence to human intestinal epithelial cells^6,26,27^. Deletion of factors involved in adhesion to host cells greatly attenuated virulence^28–32^. The ability to escape immune clearance is also necessary for strain survival, dissemination and pathogenesis. For example, CPS has been shown to enhance bacterial resistance against killing by host phagocytes^33^.

In this study, a human *S. suis* LSM178 with serotype 2 and a novel ST1005, which causes fever, nausea and general malaise, was isolated and comprehensively assessed as a hyper-virulent strain based on toxicity tests, a zebrafish model of infection and Caco-2 cell toxicity and adhesion/invasion assays and genomic analysis. With genome sequencing, genetic features including virulence factors, pathogenicity islands (PAIs), prophages and core virulence genes were characterized to understand the pathogenic potential of the strain. The analysis suggested that genome plasticity contributes to virulence evolution of *S. suis*, and specific elements predict the virulence change and adaptation of *S. suis* to humans.

## Results

### Zebrafish challenge

**Figure 1:**
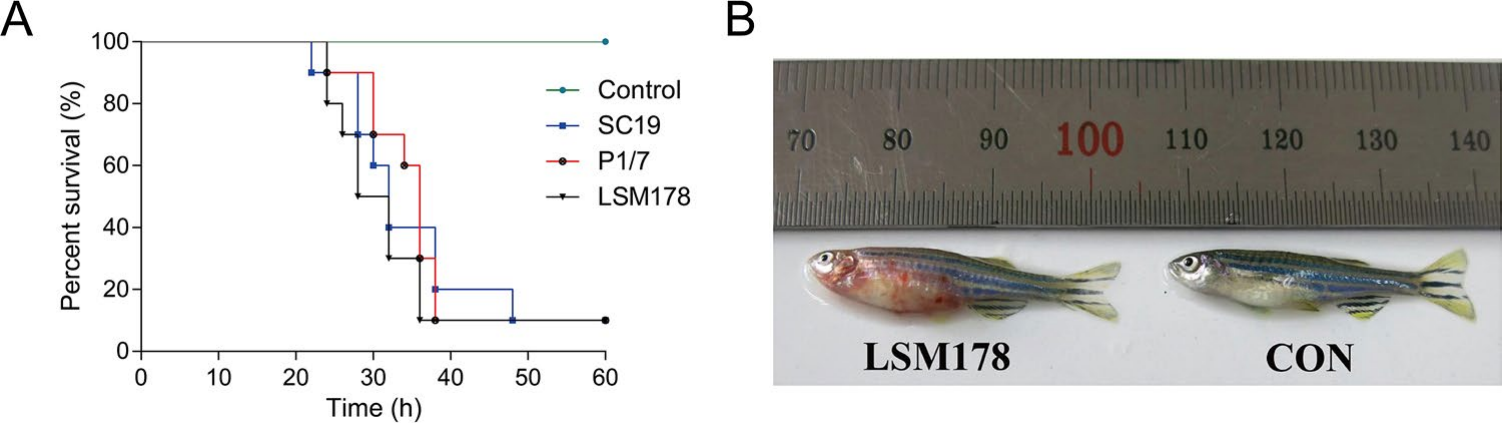
Evaluation of the virulence of the LSM178 in zebrafish. (**A**) Survival rate of zebrafish infected with LSM178, SC19 and P1/7. (**B**) Images of zebrafish infected with LSM178. Zebrafish injected with PBS were used as the control. Each group contained 10 zebrafish.

The newly isolated strain LSM178 was compared for virulence in a zebrafish intraperitoneal infection model with two other well characterised *S. sui*s isolates, SC19 and P1/7. Before 10 h, all three infection groups (LSM178, SC19 and P1/7) of infected zebrafish showed no signs of disease except for a slight decrease in swimming ability. After that, pathological changes appeared in challenged zebrafish such as systemic hemorrhage or abdominal hemorrhage, abdominal swelling and dyspnea. The survival rate did not show significant difference (*P* = 0.5287) and stabilized at 10% for three groups (Fig. 1A). However, the 50% and 90% lethal time by LSM178 (24–28 h and 34–38 h) appeared always shorter than that by SC19 (26–32 h and 38–48 h) and P1/7 (30–36 h and 36–40 h) in each of 3 independent repeated tests (Fig. 1A and Supplementary Fig. 1). The main symptoms of the death caused by LSM178 infection are serious abdominal swelling, blood spots and ecchymosis (Fig. 1B), which were similar to those caused by SC19 and P1/7.

### Human whole blood resistance assay, cytotoxic assay and cell adhesion and invasion test

**Figure 2:**
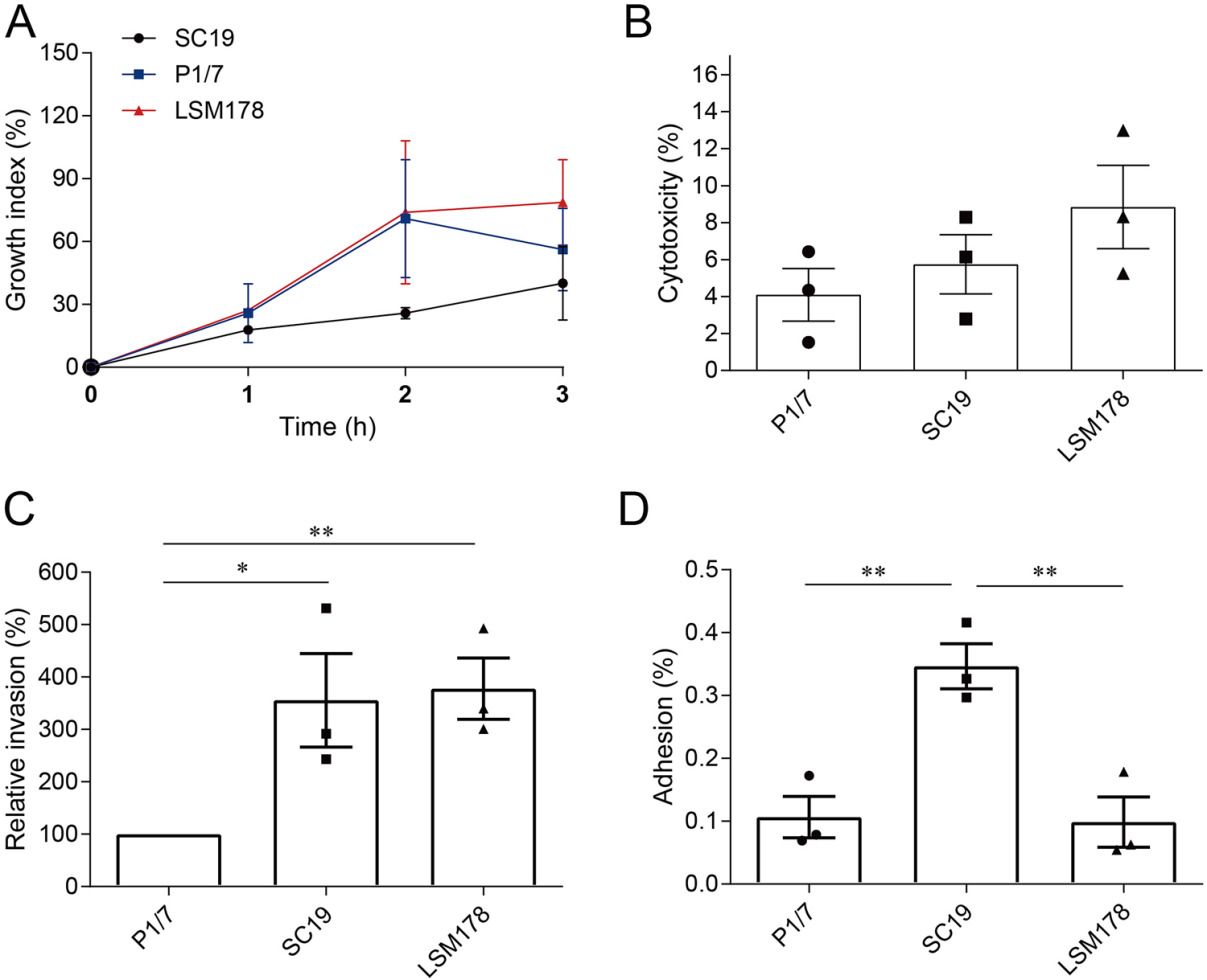
The resistance of LSM178, SC19 and P1/7 to human whole blood and expriments with Caco-2 cells. (**A**) The growth index of LSM178, SC19 and P1/7 in human whole blood. (**B**) The cytotoxicity of LSM178, SC19 and P1/7 to Caco-2 cells. (**C**) The relative invasion of LSM178, SC19 and P1/7 to Caco-2 cell. (**D**) The adhesion of LSM178, SC19 and P1/7 to Caco-2 cell. Invasion and adhesion were performed 3 h post infection. The data point represents the average of experimental repeats. *P* < 0.05 *, *P* < 0.01 **.

In a human blood resistance assay, LSM178 survived similarly to P1/7 (*P* = 0.7948), but seemed grow better than SC19 (*P* = 0.2905) (Fig. 2A). Although there was no significance in cytotoxity to Caco-2 among the three strains, LSM178 appeared more cytotoxic than SC19 (*P* = 0.3244) and P1/7 (*P* = 0.1492) and was almost twice as toxic as P1/7 strain (Fig. 2B). Further, LSM178 showed significantly more invasion of Caco-2 cells than P1/7 (*P* = 0.0090), but similar to SC19 (*P* = 0.8454) (Fig. 2C). Both LSM178 (*P* = 0.0099) and P1/7 (*P* = 0.0079) had significantly lower adhesion to Caco-2 cells than SC19 (Fig. 2D).

### Genomic features

**Figure 3:**
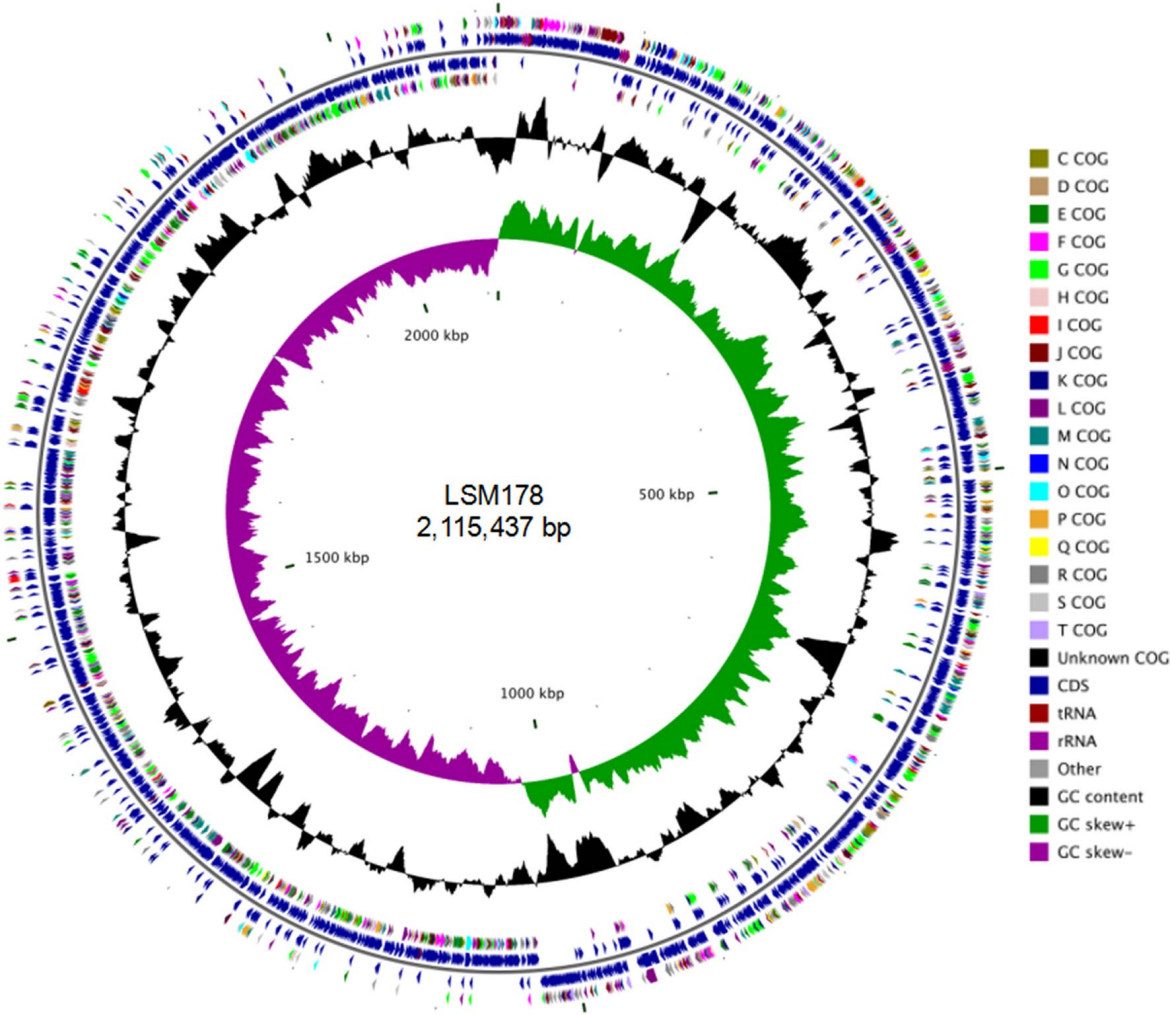
The circular diagram of the LSM178 genome. From inside to outside, the first circle, the scale of genome; the second circle, GC skew; the third circle, GC content; the fourth and seventh circles, the COG category of coding sequence (CDS) on two strands; the fifth and sixth circles, the position of CDS, tRNA and rRNA on two strands. The image was generated by online website CGView Server BETA (http://cgview.ca/).

The LSM178 genome (accession number, CP047248) consists of a single circular chromosome of 2,115,437 base pairs (bp) with average GC content of 41.19% and 2,065 ORFs (Fig. 3). Sequence analysis designated LSM178 with serotype 2 and a novel ST (1005). With goeBURST analysis, ST1005 was shown to be an individual ST (Supplementary Fig. 2).

### Analysis of antimicrobial susceptibility profiles

The scanning results showed that LSM178 possesses 10 potential specific antibiotic-resistant genes. Only two of these corresponded to a resistance phenotype in antimicrobial susceptibility tests, a tetracycline resistant gene (*tetM*, 04,995) conferring the tetracycline resistance and a MSL methylase (04,940) encoding gene resulting in both erythromycin and clindamycin resistance (Table 1). Other potential antibiotic-resistance genes include those encoding five penicillin-binding proteins (PBPs, PBP1b (00,760), PBP1a (02,025), PBP1a (09,665), PBP2b (03,000) and PBP2x (08,260)), an Aminoglycoside 6-adenylyltansferase (ANT(6)-Ia, 04,805) and two primary targets of quinolone (DNA gyrase (GyrA, 04,275) and topoisomerase IV (ParC, 05,695)). These are ineffective since LSM178 was sensitive to penicillin, streptomycin and levofloxacin (Table 1).

**Table 1:** Minimal inhibitory concentration (MIC) of 12 antimicrobial agents for LSM178 and the resistance phenotype (RP).

	Antibiotics MIC (μg/mL)											
	PEN	TET	VAN	CRM	CHR	CLI	LVX	ERY	STR	IPM	LZD	RFP
ATCC 49,619	0.25	≤ 0.5	≤ 0.12	0.5	2	≤ 0.06	0.5	≤ 0.06	0.12	0.12	≤ 0.06	0.25
LSM178	≤ 0.06	> 3	0.25	≤ 0.25	< 3	> 0.5	0.75	> 0.5	≤ 0.12	≤ 0.06	0.12	0.25
RP	−	+	−	−	−	+	−	+	−	−	−	−

*Abbreviations* PEN, penicillin; TET, tetracycline; VAN, vancomycin; CRM, cefuroxime; CHR, chloramphenicol; CLI, clindamycin; LVX, levofloxacin; ERY, erythromycin; STR, Streptomycin; IPM, Imipenem; LZD, Linezolid; RFP, rifampicin; + , resistance; − , sensitivity.

### Phylogenetic tree analysis

**Figure 4:**
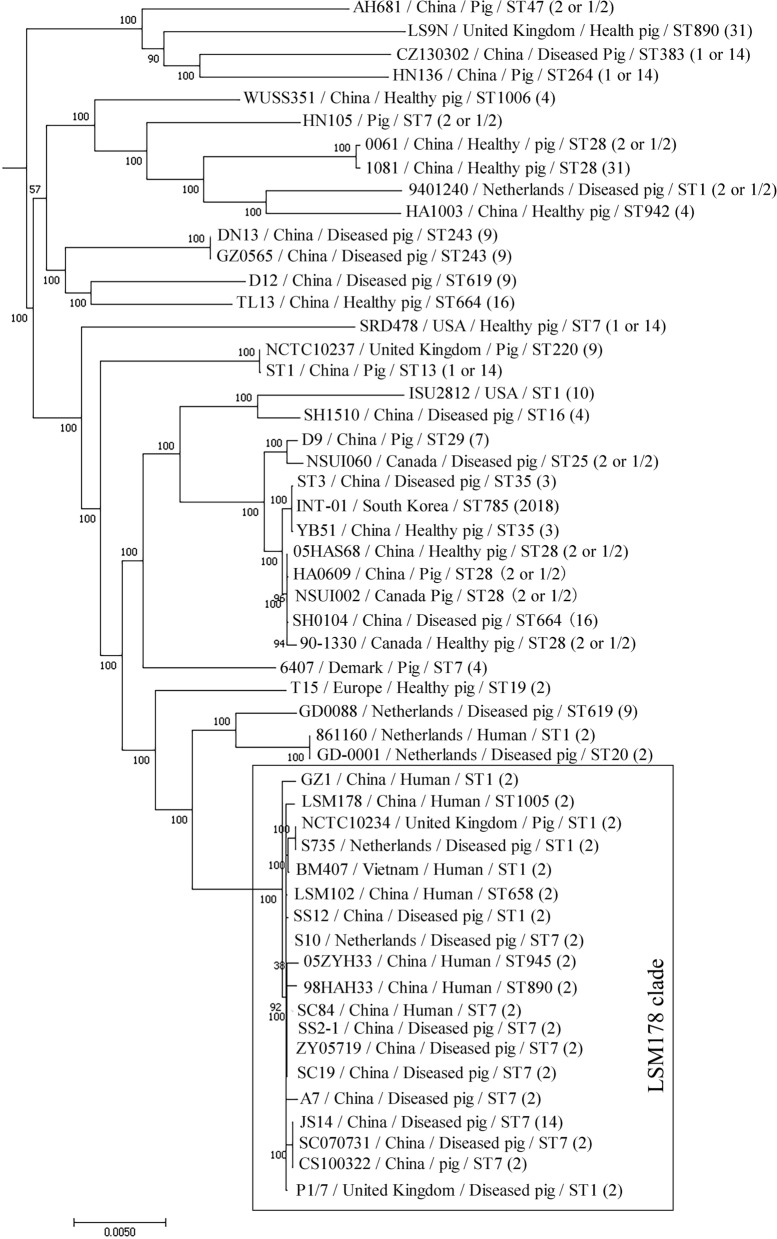
Phylogenetic tree of 53 *S. suis* based on core genome SNP. The image was generated by FastTree embedded in the Parsnp v1.1.2 and online website iTOL (http://itol.embl.de/).

Using the genome sequence of LSM178 and 52 published *S. suis* complete genomes, a phylogenetic tree was generated with the 51,520 core-genome SNPs from the nonrecombinant regions (Fig. 4). Within the 19 strains clustered with LSM178 (LSM178 clade), 7 out of 12 pig isolates and 6 out of 7 human strains were from China. Among the 53 strains, most ST1 (6/9) and ST7 (9/12) were clustered in LSM178 clade. It was interesting that in LSM178 clade, the STs of human isolates (GZ1, LSM178, LSM102, 05ZYH33, SC84, BM407 and 98HAH33 with ST1, ST1005, ST658, ST945, ST7, ST1 and ST890 respectively) are more diverse than those of pig strains (just including ST1 and ST7).

### Analysis of prophages and toxin-antitoxin (TA)

**Figure 5:**
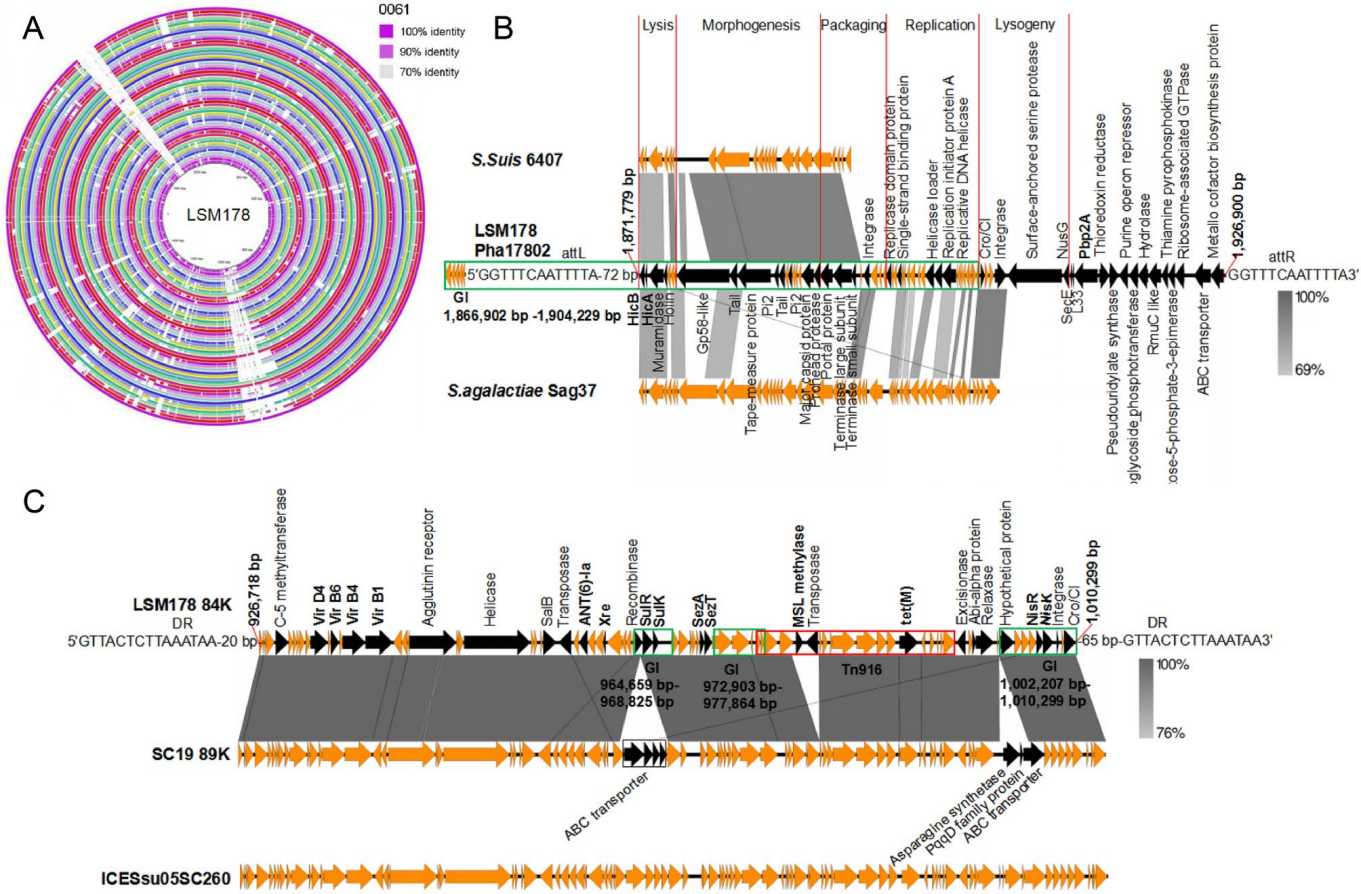
Genome analyse of LSM178. (**A**) Genome comparison among 52 *S. suis* and LSM178. Each circle showed the variations of strain relative to LSM178. From inside to outside, the genomes was as follows: 0061, 05HAS68, 05ZYH33, 1081, 6407, 861,160, 90–1330, 9,401,240, 98HAH33, A7, AH681, BM407, CS100322, CZ130302, D12, D9, DN13, GD-0001, GD-0088, GZ0565, GZ1, HA0609, HA1003, HN105, HN136, INT-01, ISU2812, JS14, LS9N, LSM102, NCTC10234, NCTC10237, NSUI002, NSUI060, P1/7, S10, S735, SC070731, SC19, SC84, SH0104, SH1510, SRD478, SS12, SS2-1, ST1, ST3, T15, TL13, WUSS351, YB51 and ZY05719. The variable colors in each circle stand for sequence identity with the inset of *S. suis* 61 as an example. (**B**) Comparison of the 84 K-PAI with the 89 K-PAI from SC19. The 84 K-PAI were marked with key genes (black) including virulence-related factors (bold), antibiotic resistance factors (bold), major differential genes and core transposition elements. Green box, GIs; Red box, Tn916. (**C**) Comparison of the pha17802 with the homologous region located in *S. suis* 6407 and *S. agalactiae* Sag37. Genes rather than hypothetical protein-coding ones were given predictable functions (black). Green box, GI. The functional regions were designed with red line. The image of A was generated using BLAST Ring Image Generator v0.95 (BRIG). The images of B and C were generated using BLAST embedded in Easyfig v2.25.

Two prophages, pha17801 (positions 1,417,367–1,458,272) and pha17802 (positions 1,871,779–1,926,900), were found in LSM178. The pha17801, with 50 ORFs and GC content of 39.36%, contains two integrases (positions 1,437,316 − 1,437,858 / 1,446,215–1,447,357) and is highly similar to the partial genome of *Streptococcus* phage 20,617 (RefSeq accession NC_023503). The prophage included two overlapping GIs (positions 1,421,901–1,447,702 and positions 1,437,316–1,447,702). These two GIs were shared only by 18 *S. suis* (LSM178, 05ZYH33, 98HAH33, A7, BM407, CS100322, GZ1, JS14, LSM102, P1/7, S10, SC070731, SC19, SC84, SS12, SS2-1, T15 and ZY05719) (Fig. 5A). Excluding avirulent T15^34^, these strains almost constituted the LSM178 clade (Fig. 4).

In pha17802, the overlapped GI (positions 1,866,902–1,904,229) was characteristic to LSM178 in comparison with other 52 *S. suis* (Fig. 5A). The left part (positions 1,871,862–1,892,563) of the GI is only highly similar to (identity of 90%) a region of *S. suis* 6407, whereas the right part (positions 1,892,563–1,903,867) together with sequence of positions 903,868–1,906,268 is not homologous with any *S. suis* but with *Streptococcus agalactiae* Sag37 (identity of 91%) (Fig. 5B). The prophage comprises 55,121 bp with an average GC content of 41.87%, containing a total of 68 ORFs including prophage core component genes encoding lysin, tail, head, recombinase, capsid, portal, integrase, portal, and Cro/Cl-type repressor. The absence of excisionase confirmed the non-plaques on several strains containing LSM178 (data not shown). The short directly repeated sequences (5′GGTTTCAATTTTA3′) located the prophage between 09,360 (adenylate kinase) and 09,655 (preprotein translocase subunit SecE). The sequence of positions 1,866,902–1,906,268 contained 23 function-known genes along with 29 encoding hypothetical proteins and the unique sequence (positions 1,892,563–1,906,268) involved 29 genes where 21 encode unknown proteins. The TA of HicA (09,375)-HicB (09,370) is the only candidate virulence elements in this phage. Other TAs were listed in Supplementary Table 1.

### Comparative analysis of virulence factors

Examining the 96 virulence factors among 53 *S. suis* genomes, LSM178 is one of the 8 strains (LSM178, ZY05719, 98HAH33, SC84, 05ZYH33, SS2-1, SC19 and LSM102) containing the most (95) virulence factors (Supplementary Table 2). The 95 factors contain 15 linked to immune evasion or systemic infection, including SalKR, NisKR, Epf, Fhb, IgA1, Ide_*S. suis*_, MRP, Sly, Nudp, SsnA, EndA, ScpA and SsadS. Excluding GZ1 and BM407 with 87 and 93 virulence factors respectively, these 8 strains are all human and/or epidemic strains. The Rgg is the only virulence factor missing in the 8 *S. suis* and it is found only in 5 other *S. suis* (D12, 0061, 1081, CZ130302 and HN105). The common characteristics of virulence factors in the 8 highly virulent strains could explain the highly pathogenic phenotype of LSM178.

An 84 kb PAI (84 K-PAI) (positions 926,718–1,010,299) was found to be highly similar to type I-89 K-PAI of SC19 and both of them are also similar to ICESsu05SC260 belonging to ICESa2603 family (Fig. 5C). The direct repeat sequences indicated the location of the 84 K-PAI just downstream of *rplL* (04,665). The PAI encodes characterized virulence factors such as SalKR, NisKR and several type IV secretion systems (Vir D4, Vir B6, Vir B1 and Vir B4). The presence of integrase, transposase, excisionase and helicase support activity of transposition and propagation of 84 K-PAI. The major difference of LSM178 84 K-PAI from SC19 89 K-PAI is the absence genes encoding PqqD family protein, asparagine synthetase and 4 ABC transporter units, and addition of genes encoding transposase and MSL methylase as described. Apart from the methylase which may cause epigenetic changes associated with virulence, the encoded proteins are not defined as virulence factors and should not contribute to virulence.

The 89 K-PAI has evolved to be diverse^22^. The type I-89 K-PAI was first identified in SS2-1 (diseased pig, 1998) and 98HAH33 (human, 1998), close relatives of SC84 (human, 2005), ZY05719 (pig, 2005) and SC19 (pig, 2005) (Fig. 5A and Fig. 4). From 2005, the PAI was reported in almost all human strains (SC84, 2005; 05ZYH33, 2005; LSM102, 2014 and LSM178, 2016) apart from GZ1 (2005), but only in two (ZY0571, 2005 and SC19, 2005) rather than the other 10 pig strains. The human strains not from China (861,180/Netherlands/1986 and BM407/Vietnam/2004) do not contain type I-89 K-PAI (Figs. 4  and 5A). The distribution of the type I-89 K-PAI among the strains coincides interestingly with the combination of the most (95) virulent factors (Fig. 5A and Supplementary Table 2). Since LSM178 shares these important virulence markers with epidemic strains and almost all human strains, these strains were referred here as the highly virulent strains (HVS) at least for serotype 2 strains from China, those are LSM178, ZY05719, 98HAH33, SC84, 05ZYH33, SS2-1, SC19 and LSM102.

### Genomic comparative analysis

**Figure 6:**
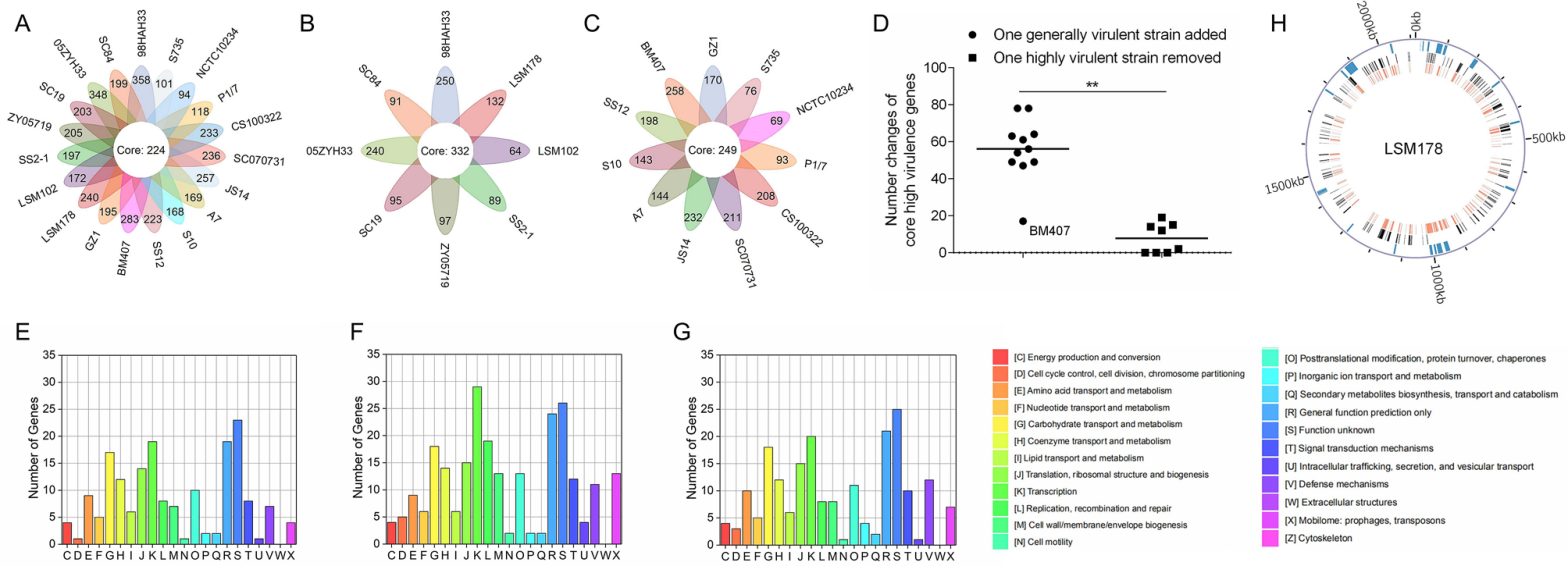
Chromosomal features of LSM178. (**A**) Venn diagram of the special genes of each strain from LSM178 clade. The special genes for each strain were extracted with T15 as the reference. The overlap was named core virulence genes. (**B**) Venn diagram describing the special genes of HVSs. (**C**) Venn diagram describing the special genes of generally virulent strains. (**D**) Changes of core high virulence genes. *P* < 0.01 **. (**E–G**) COG function classification of the core genes from (**A–C**) respectively. (**H**) Locations of GIs (blue), core general virulence genes (black) and core high virulence genes (red) in LSM178 genome. The images of **A**, **B** and **C** were generated using RStudio v4.0.5. The image of H was generated using circos v0.69. The images of E, F and G were generated using Origin 2019.

Comparison of virulence factors between strains in LSM178 clade and closely related avirulent T15 identified 87 virulence factors which were shared by T15 and the LSM178 clade strains, except A7, P1/7, S10 and GZ1 (Supplementary Table 2). These four strains had 87 virulence factors, but they share Epf, NadR, RevS and SBP2 instead of Trag, VirB1, VirB4 and VirD4 as in T15. However, there were no virulence factors belonging exclusively to avirulent T15 or all LSM178 clade strains. To globally identify the characteristic virulence factors, the difference was checked between strains in LSM178 clade and closely related avirulent T15. Referring to T15, the numbers of unique genes of each virulent strain were between 318 and 582, and 224 shared genes were found (Fig. 6A). HVSs possess more core genes (332) than other virulent strains (249, called generally virulent strain here) (Fig. 6B and C). Addition of any one generally virulent strain except BM407 (Vietnam) resulted sharp decreased number of core high virulence genes (Fig. 6D). However, little change was observed when any one highly virulent strain was removed. This supports HVSs as a seperate group in serotype 2 strains. The characteristic genes from various categories encode a wide range of functions (Fig. 6E–G). Compared with generally virulent strains, HVSs show predominant increase on genes with functions of transcription, cell barrier, replication, recombination, repair and mobile elements (Fig. 6F and G). GIs of LSM178 occupy 10.4% of the genome (Fig. 6H). While there are 3.7% of core virulence genes in GIs of LSM178, 23.5% of core high virulence genes locate in the GIs (Fig. 6H).

## Discussion

Generally, *S. suis* infections in humans are restricted to workers in close contact with pigs or swine byproducts. However, in southeast Asia, the bacterium has been reported to affect the general population^23^. The sporadic human *S. suis*, particular those with serotype 2, should get attention in epidemiological monitoring because of their unpredictable adaptive potential, as demonstrated by LSM178, a serotype 2 strain with a novel ST1005. LSM178 was more efficiently invasive to Caco-2 cells compared with P1/7 and SC19. However, no significant virulence was detected in cytotoxic activity and zebrafish challenge assays which have been used as a model to evaluate the virulence of *S. suis*^35^. Indeed, virulence of *S. suis* could not be intensively evaluated and compared in other models^36^. Probably, virulent strains have their own outstanding virulence aspects, which can balance the weak ones and eventually lead to a similar pathogenicity at least under the inoculation. For instance, P1/7 and SC19 were prominent in biofilm formation (Supplementary Fig. 3) and adhesion respectively.

Recent studies suggested that quinolones, beta-lactams, florfenicol and trimethoprim/sulfamethoxazole could still effectively treat clinical *S. suis* infection^37–39^. However, strains resistant to widely used effective beta-lactams have been increasely reported^40,41^. LSM178 showed the sensitivity at least to beta-lactams and quinolones, consistent with the features of resistance genes. The PBPs of LSM178 are entirely the same as those of sensitive A7, but habor substitutions throughout the sequence compared with the resistant R61 (Supplementary Fig. 4A–E)^40^. Several mutations in the quinolone resistance-determining region (QRDR) of both GyrA and ParC could increase the resistance to fluoroquinolone^42^. However, no amino acid changes were revealed in the QRDRs between LSM178 and four quinolone-sensitive strains (A7, BM407, P1/7 and SC84)^40^ (Supplementary Fig. 5). Additionally, it is interesting that the P1/7 without ANT(6)-Ia confered resistance against streptomycin, whereas ANT(6)-Ia containing LSM178 could not^38^. However, ANT(6)-Ia of LSM178 is only 49.5% identical to the functional homologue (UniProtKB-P12055 (AADK_STAAU)) from *Staphylococcus aureus*^43^ (Supplementary Fig. 6).

Several factors are deemed to be important for the pathogenesis, such as CPS, Fbps, enolases, dipeptidylpeptidase DppIV and SrtA^25^, which were all found in LSM178. It has been demonstrated that *S. suis* serotype 2 virulent strains are able to exacerbate inflammatory activation scavenging bacteria. All the 15 known anti-immunity factors^22^ exist in LSM178 and would modulate the immune responses improving its survival at the inflammation area. Although it is difficult to define a virulent strain only by proposed virulence genes, strains isolated from healthy animal are found with less virulence factors (less than 87), such as WUSS351, 0061, 1081, HA1003, TL13, 05HAS68, YB51, LS9N, SRD478, 90–1330 and T15. These *S. suis* lack at least one of the marker virulence genes (*sly*, m*rp*, *epf* and *cps2*)*.* Notably, four of those strains contain less than 70 virulent factors (0061, 1081, HA1003 and LS9N). On the contrary, the HVSs contain consistently the most (95/96) virulent factors. However, it should be realized that the combination of various virulence factors may cause pathogenicity despite the number of the virulence factors. For instance, the CZ130302 contains 69 virulence factors but shown to be a virulent strain^11^, and the strains other than T15 isolated from healthy animal are not necessarily the avirulent strains.

It is very clear that intermediately pathogenic strain could evolve to become a highly pathogenic and then epidemic strain^4^. It should be noted that the evolutionary relationships could be lack of reliably based on the raw sequence due to the extensive genetic recombination in *S. suis*^24^. With the nonrecombinant regions, phylogenetic analysis should show the reliable evolutionary relationships^1^. In LSM178 clade, all 19 strains possessed the serotype 2. However, while 7 human strains have 6 STs, 12 pig strains were just ST1 (4 strains) or ST7 (8 strains). It may imply that while *S. suis* has been purified to relatively stable genotype in pig, multiple evolutionary directions are in progress to be epidemic during their adaption to human, at least for serotype 2 strains in China.

Type I-89 K-PAI is specific to highly pathogenic *S. suis* linked to Chinese epidemics^22^ and could horizontally transfer among strains^44^. Humans can carry avirulent *S. suis* without clinical signs^3,45^. However, the 89 K-PAI of human virulent strain should not be obtained from pig strain owning to the transfer, since all strains with 89 K-PAI presented an extremely short evolutionary distance from each other. It suggested that human pathogenic infection was due to the interspecies transmission of a swine-origin strain. The gradual increase of 89 K-PAIs in human strains and decrease in pig strains may suggest that the 89 K-PAI is one of the markers adapting pig strain to human. On the contrary, the existence of PAI would not be conducive to the adaptation of strains in pigs, that might explain the reason why the pig strains lose it more and more rapidly. Under this consideration, the combination of 95 virulence factors may also be regarded as indicative one of the potential for the adaptation to human.

In published *S. suis* genomes, many prophages were held as remnants^46,47^. A few intact prophages was described and one of them was reported to be induced to lyse *S. suis*^48^. The two prophages in LSM178 could not be induced to form plaques on several *S. suis* strains. The reason might be that improper induction method was adopted or that the phages produced are defective on infection even for the almost intact pha17802^48^. Alternatively, the lysis spectrum of the phages is very narrow, or no phages were induced at all, which could be supported by the facts that pha17801 contains only integrase and pha17802 does not include excisionase. Genomic comparative analysis suggested that the pha17802 may integrate two elements horizontally transferred from *S. suis* 6407 and *S. agalactiae* Sag37 respectively. The unique gene fragment from *S. agalactiae* Sag37 has not been found in any other *S. suis* genome published so far. The empty target site might accommodate potentially unpredictable integration of other mobile genetic elements. Prophages make up a platform for the dissemination of virulence determinants between intra- and inter-species, contributing to the evolution of pathogenic bacteria^30,49^. For pha17802, except the *hicAB* located in the arm homologous to 6407, no known genes contributing to virulence were identified. There was no evidence demonstrating the contribution of the two transferring elements to virulence. However, the rarity of pha17802 suggested that it might provide an advantage under certain circumstances. At least, the lysogenic state would increase the survival in the environment by resisting to infection of similar viruses. Furthermore, it may increase the fitness of the bacteria by modulating host metabolism^48^.

While the core virulence genes were identified through the comparation between avirulent T15 and virulent strains in LSM178 clade, they should be responsible for the enhanced pathogenicity. Moreover, some genes could be used to make a distinction between HVSs and other general virulent strains. Particularly, it should emphasize the roles of genes with functions of transcription, cell barrier, replication, recombination and repair in virulence enhancement, since their number increased obviously in HVSs core genes. Thus, the importance of the mobilizable elements is beyond all doubt, because it is the carrier responsible for genetic differences^50^. In fact, GIs make up a considerable part of the genome for LSM178 (10.4%). And, higher proportion of virulence-enhancing genes are dispersed in GIs, for example that GIs of LSM178 are colonized with 3.7% of core general virulence genes but with 23.5% of core high virulence genes. However, there are less GIs in LSM178 (number of 23 and total length of 21, 5087 bp) than T15 (29 and 31, 4245 bp) (Supplementary Fig. 7B). These suggested that fusion of specific PAIs increased the virulence. The non-existence of CRISPRs defending against foreign invading elements should be one of the reasons interpreting the rich GIs^51^. In addition, assay showed that there are more core avirulence genes (535) (Supplementary Fig. 7A) than core virulence genes (224). Probably, both the gain of virulence genes and loss of avirulence genes contribute to the increased virulence. Other potential difference, such as SNPs and patches of insertion and deletion, deserve also to be explored and should not be ignored. These differences are small but numerous and they would definitely interpret the change of strain virulence through just affecting the genes expression or protein activity^52^.

## Materials and methods

### Strains and antimicrobial susceptibility testing

*S. suis* LSM178 was isolated at 2016 from a patient with clinical symptoms of fever, nausea, and general malaise. The patient had been in contact with pigs and handled raw pork before admission. The *S. suis* SC19 and *S. suis* P1/7 were stored in our laboratory. Antimicrobial susceptibility was tested by E-test (AB Biodisk, Sweden) with *Streptococcus pneumoniae* ATCC49619 as a control. All protocols was approved by committee of State Key Laboratory of Agriculture Microbiolgy and the ethics committee of Huazhong Agricultural University. Experiments were performed in ABSL 3 laboratory, Huazhong Agricultural University.

### Human whole blood resistance assay

Blood assays were conducted according to an approval issued by the Medical Ethics Committee of the Huazhong Agriculture University (Wuhan, China). Strain suspension (100 μL, 5.0 × 10^7^ CFU/ml) was transferred into 900 μL of fresh human whole blood followed by incubation at 37℃. Samples were withdrawn every 1 h and diluted to incubate on TSA solid medium (containing 10% fresh FBS) at 37 ℃ overnight to count colony-forming units (CFU). Growth index (%) = (CFU_at a certain time point_—CFU_original inoculum_) / CFU_original inoculum_ × 100%.

### Zebrafish challenge

Zebrafish were fed as previously described^53^. Inoculum was collected at the end of the logarithm period, cleaned twice with phosphate buffered saline (PBS), and adjusted to the appropriate dose (2.5 × 10^9^ CFU/ml)^35^. Adult zebrafish were infected by intraperitoneal inoculation with 20 μL of bacterial solution per tail. Each group contained 10 zebrafish. The symptoms of zebrafish were recorded every 2 h. Euthanasia of zebrafish was conducted at 60 h after challenge using Tris-buffered tricaine at a concentration of 320 μg/ml.

### Biofilm formation assay

Strains (20 μL, 5.0 × 10^7^ CFU/ml) were inoculated into 2 mL TSB medium (containing 10% fresh FBS) and cultured in a 24-well cell plate at 37℃ for 3 days, and the un-inoculated medium was used as the control. After that, the strains were washed twice with sterilized PBS, and fixed with 500 μL methanol for 30 min to attach the strains to the wall. Then, methanol was removed and the plate was air-dried at room temperature. Next, 500 μL of 0.1% crystal violet dye solution was added in and removed out until 30 min later. After drying at 56℃, 500 μL of 33% acetic acid solution was added and placed on a shaker for 30 min to release the crystal violet bound to the biofilm. The released solution (200 μL) from each well was measured at a wavelength of 600 nm.

### Cell experiments

Strains at log phase was used in the expriments. For cytotoxic assay, Caco-2 cells (1 × 10^4^)^54^ in 96 well plate was used to detect cytotoxicity of the strains (2 × 10^5^ CFU) with lactate dehydrogenase kit (Beyotime, Beijing, China). The percentage of cytotoxicity was calculated referring to the protocol of the kit: cytotoxicity (%) = (LDH release from infected cells –spontaneous release of LDH from uninfected cells) / (maximum LDH release from cell lysate–spontaneous release of LDH from uninfected cells) × 100%.

For cell adhesion and invasion, single layer Caco-2 cells in the 24 well culture plate were inoculated with 500 μL bacterial suspension (1 × 10^6^ CFU) for 3 h. After washing to remove unadhesive strain, cells were then treated by trypsin digestion for 2 h. In invasion, extracellular bacteria were treated with gentamicin (100 μg/ml) and penicillin G (5 μg/ml) before trypsin treatment. The digested cells were lysed using 1% saponin and the lysis was inoculated on THB plate. The rate of adhesion (Ra) and invasion (Ri) was expressed as (CFU _determined from plate_ / CFU _original inoculum_) × 100%. The relative invasion rate was expressed as Ri _strain_ / Ri _P1/7_ × 100%.

### Plaque assay

LSM178 culture in exponential growth phase was induced by mitomycin C (500 ng/ml) (Sigma, St. Louis, USA) for 5–15 min. The culture (100 μL) was mixed with 3 ml TSA (45 ℃) containing 10% fresh FBS to prepare sandwich plaque assay at 37 ℃. Plaque formation was observed after 12 h.

### Genomic analysis

The genomic DNA was extracted using a DNA extraction kit (TaKaRa DNAiso, TaKaRa Biotechnology Co., Ltd., Dalian, China). The genome of LMS178 was sequenced using combined plaforms of Illumina Miniseq and PacBio sequel. Illumina Miniseq generated 4,234,826 reads producing a total of 926,438,020 bp with Q30 of 76.81%. PacBio sequel generated 234,808 reads and a total sequence length of 1,538,801,765 bp with N50 of 9,236 bp. The data from Illumina Miniseq and PacBio sequel were assembled by A5-Miseq v20150522^55^ and CANU^56^ respectively. After correction of the results using Pilon^57^, the complete circular genome was constructed.

Local virulence factor database of *S. suis* was established (Supplementary Table 2) and used to detect the potential virulence genes in genome. Of the 96 virulence factors, 84 are from the 2016 study by Willemse et al^58^. Another 12 putative virulence factors were identifed by systematic search using the term ‘*Streptococcus suis* virulence factor’ on July 27^th^ 2017 in NCBI PubMed. TAs were predicted by Rasta^59^ and TAfinder^60^. Antibiotic resistance genes were predicted with the comprehensive antibiotic resistance database (CARD) with default settings^61^. The ST was determined using the MLST typing scheme (https://pubmlst.org/ssuis/). Using BLAST (evalue ≤ 1e-10) ^62^, serotyping strategy was executed based on the levels (≥ 80%) of both identity and coverage between WZY amino acid sequence of the LSM178 and 33 standard strains with known serotypes^63^. The sequence of CpsK was used to discriminate the serotype 2 (W161) from 1/2 (C161) for all strains with serotype 2 or 1/2^64,65^. ST complexes were analysed by goeBURST^61^ program (http://goeburst.phyloviz.net). Prediction of gene islands (GI) and prophages were performed using IslandViewer 4^66^ and PHAST^67^ respectively. Clustered Regularly Interspaced Short Palindromic Repeats (CRISPRs) were predicted by CRISPR recognition tool (CRT)^68^. Open reading frames (ORFs) were predicted with Glimmer3.0^69^. tRNA and rRNA were predicted with the Aragorn^70^ and RNAmmer^71^ embedded in Prokka^72^. The proteins in prophage and PAI were integrally annotated by databases of NR^73^, eggNOG^74^, KEGG^75^, Swiss-Prot^76^ and GO^77^. Genome mapping with information was generated by CGView^78^. To identify the unique regions in the genome of LSM178, the whole-genome sequence was used as a reference to compare with that of the other 52 strains using BLAST Ring Image Generator (BRIG)^79^. The 84 K-PAI in LSM178, 89 K-PAI in SC 19 and ICESsu05SC260 were compared to determine their similarity using BLAST embedded in Easyfig^80^. Similarly, the characteristics of the prophages in LSM178 were evaluated. The differential genes between two strains were extracted with Roary^81^ to create *Venn* diagrams and their COG functions were classified. Circos^82^ was conducted to map the distribution of GIs and interesting genes in genomes of LSM178 and T15. The.

### Phylogenetic analysis

Complete genomic sequences of 52 *S. suis* sequences were downloaded from NCBI database (https://www.ncbi.nlm.nih.gov/genome/genomes/199) (Supplementary Table 3). The chromosomal sequences were aligned using Parsnp^83^ generating core-genome single-nucleotide polymorphisms (SNPs). Phylogenetic tree based on core SNP of 53 complete *S. suis* genome (including LSM178) was constructed using maximum-likelihood phylogenetic trees by FastTree embedded in the Parsnp. In sequence alignment, recombinant regions were filtered using Gubbins v2.4.0^84^. The bootstrap value was set at 1000 times. The phylogenetic tree was displayed using the online website iTOL (http://itol.embl.de/).

### Statistical analysis

Independent determinations were performed in dupicate or triplicate and experiment was repeated at least 3 times in each group. The significance was analyzed with unpaired student’s test using GraphPad Prism 5. The bar represent the mean ± standard error of mean (sem). The *P* < 0.05 and *P* < 0.01 were represented as * and ** respectively.

### Ethics statement

This study was carried out in compliance with the ARRIVE guidelines. The study was approved by ethics committee of Huazhong Agricultural University and all experiments were performed in accordance with guidelines of State Key Laboratory of Agriculture Microbiology. The informed consent was obtained from all participants and/or their legal guardians.

### Statement on Guidelines for Human

All procedures performed in studies involving human participants were in accordance with the ethical standards of national research committee and with the 1975 Helsinki Declaration (or its later amendments).

### Statement on Guidelines for Animal

All procedures performed in studies involving animal were in accordance with the ethical standards of national institutional guidelines on the care and use of animals.

## Supplementary Information

Supplementary Information 1.

Supplementary Information 2.

Supplementary Information 3.

Supplementary Information 4.

Supplementary Information 5.

Supplementary Information 6.

Supplementary Information 7.

Supplementary Information 8.

Supplementary Information 9.

## Data Availability

The whole-genome sequence of the LSM178 was deposited in NCBI Genbank (Accession Number CP047248). Accession numbers for raw data from Illumina Miniseq and PacBio sequel are SRR15853891 and SRR15943338 respectively.
