# Supplementary figures and images for: Genome analysis provides insight into hyper-virulence of Streptococcus suis LSM178, a human strain with a novel sequence type 1005
Source: Sci Rep. 2021 Dec 14;11:23919. doi: 10.1038/s41598-021-03370-0 (PMC8671398; doi:10.1038/s41598-021-03370-0)

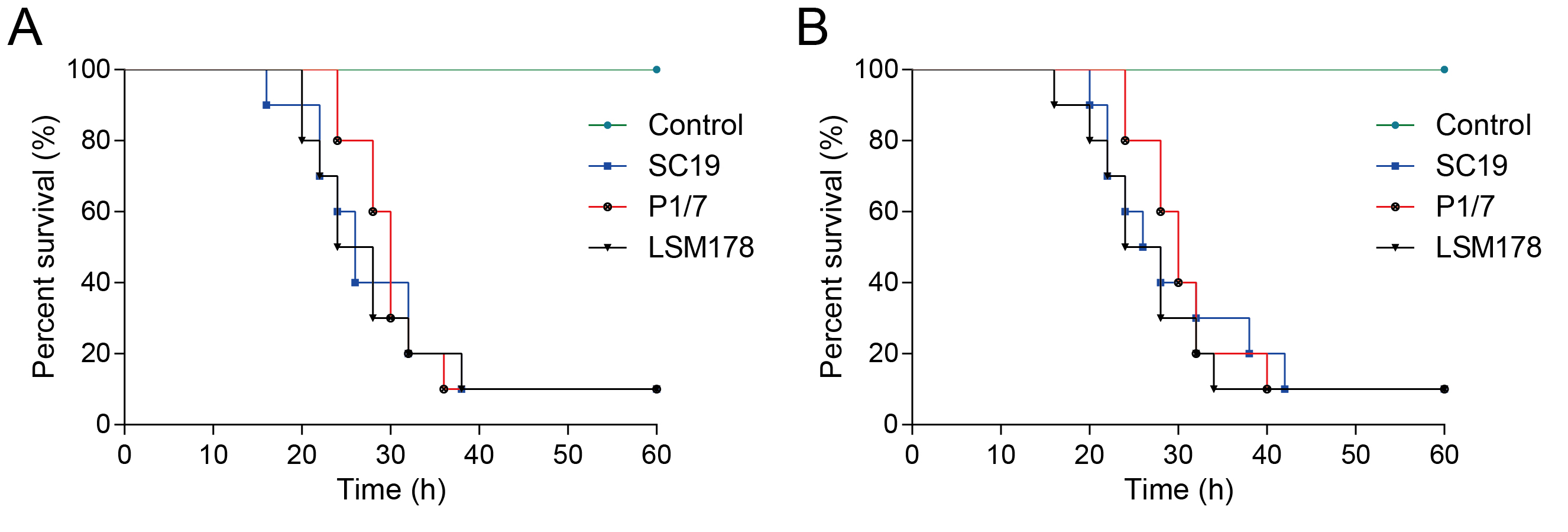

Supplement: Supplementary file 1 — Supplementary Information 1. [file 41598_2021_3370_MOESM1_ESM.jpg]

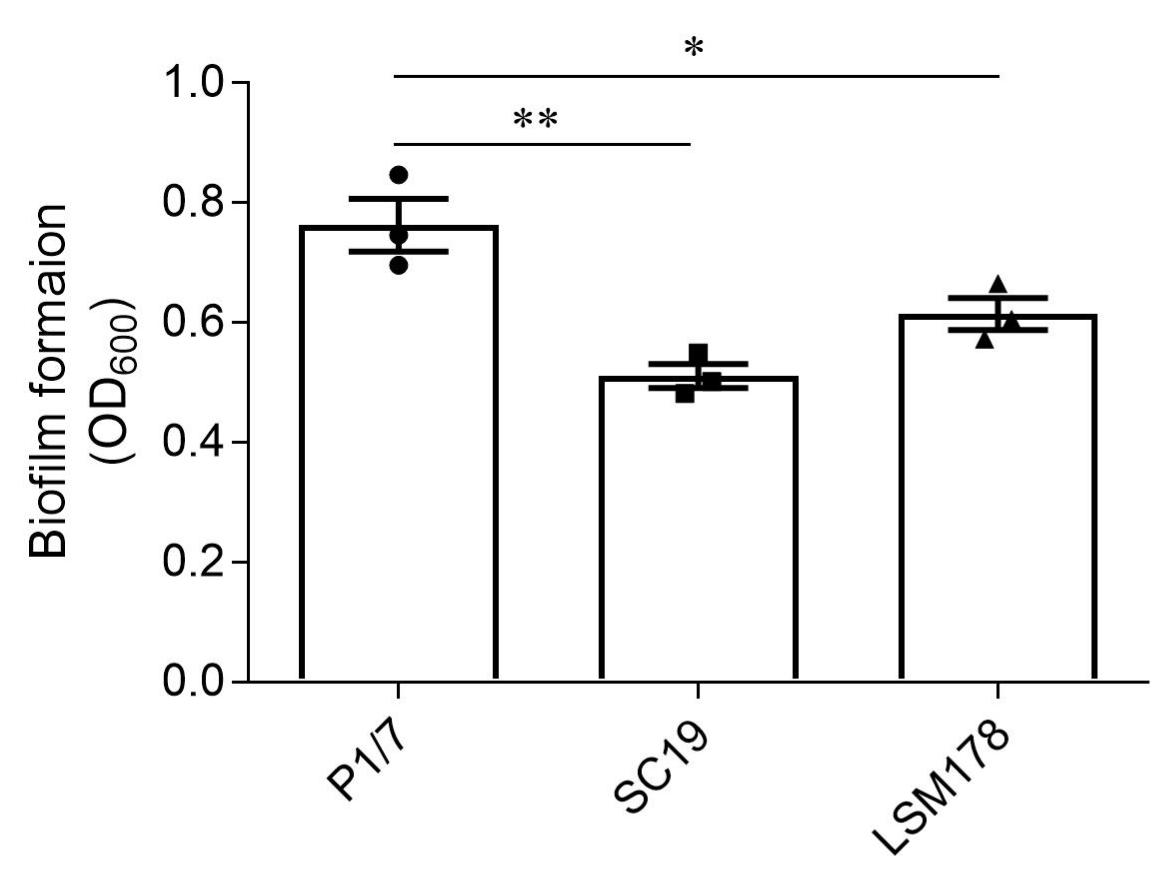

Supplement: Supplementary file 3 — Supplementary Information 3. [file 41598_2021_3370_MOESM3_ESM.jpg]

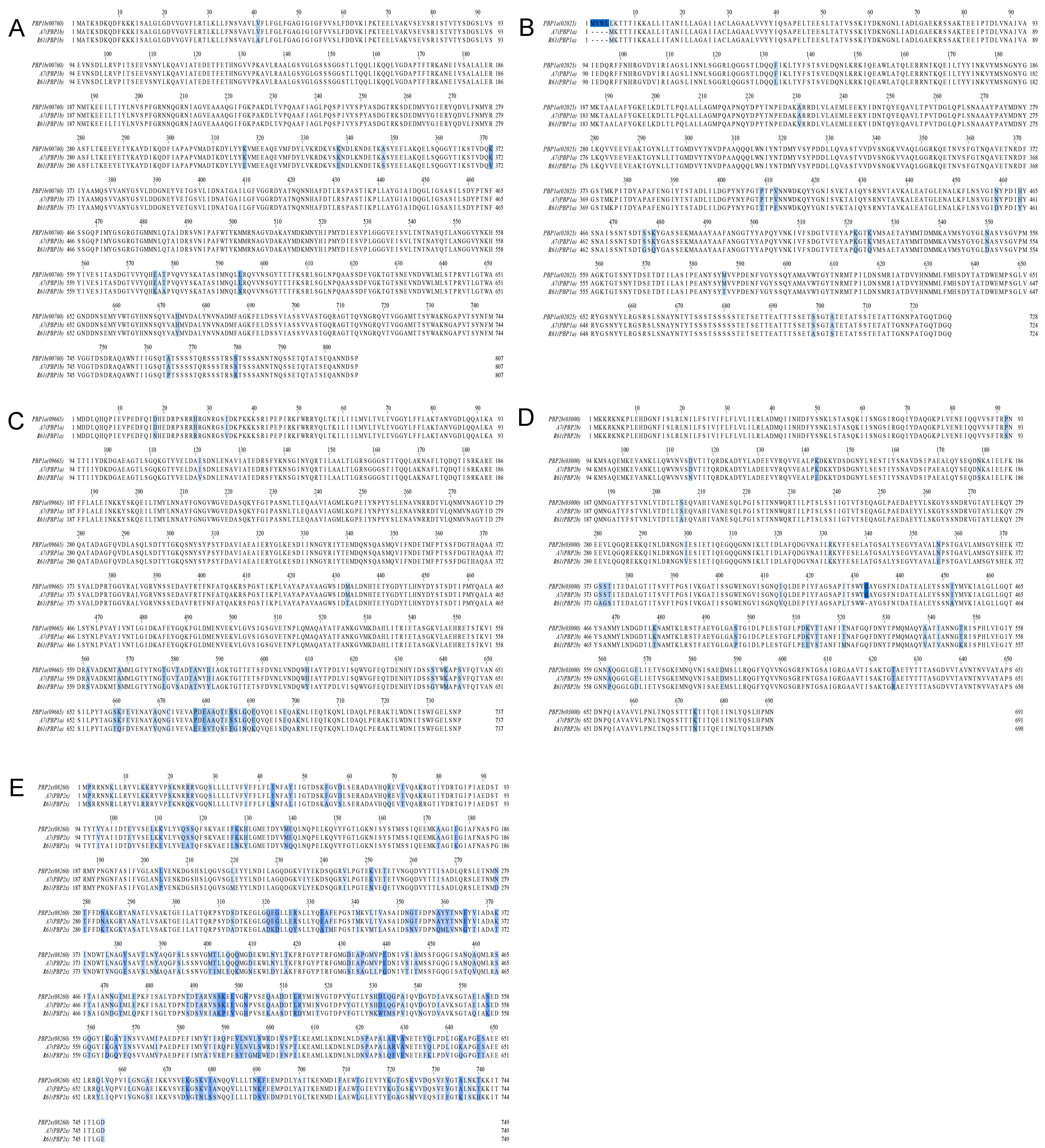

Supplement: Supplementary file 4 — Supplementary Information 4. [file 41598_2021_3370_MOESM4_ESM.jpg]

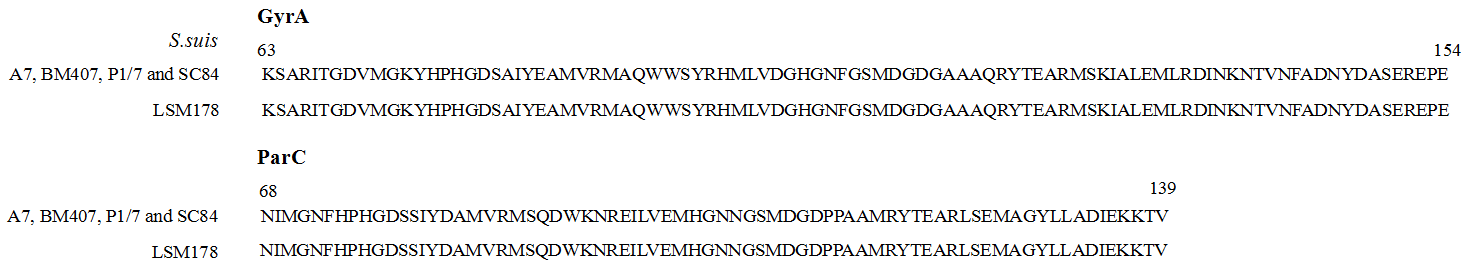

Supplement: Supplementary file 5 — Supplementary Information 5. [file 41598_2021_3370_MOESM5_ESM.jpg]

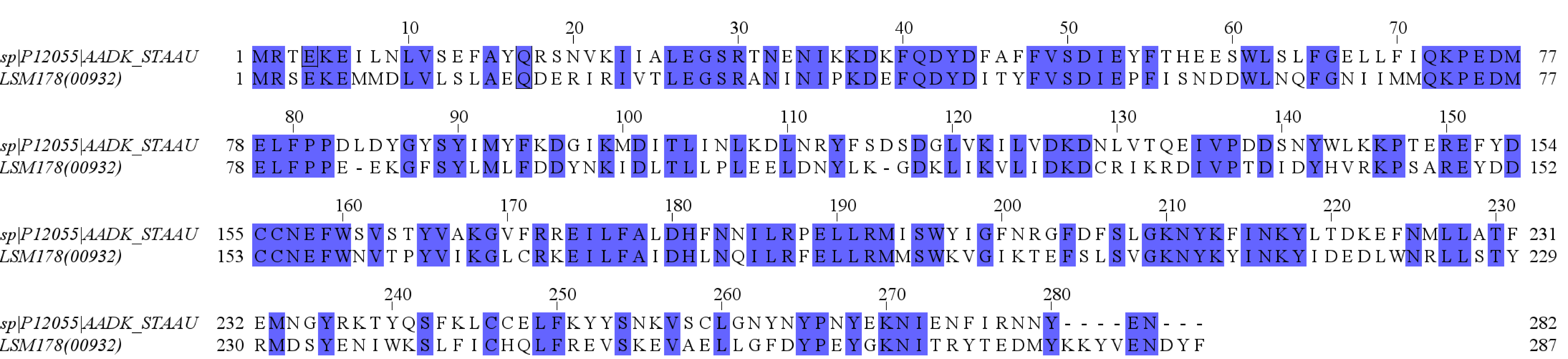

Supplement: Supplementary file 6 — Supplementary Information 6. [file 41598_2021_3370_MOESM6_ESM.jpg]

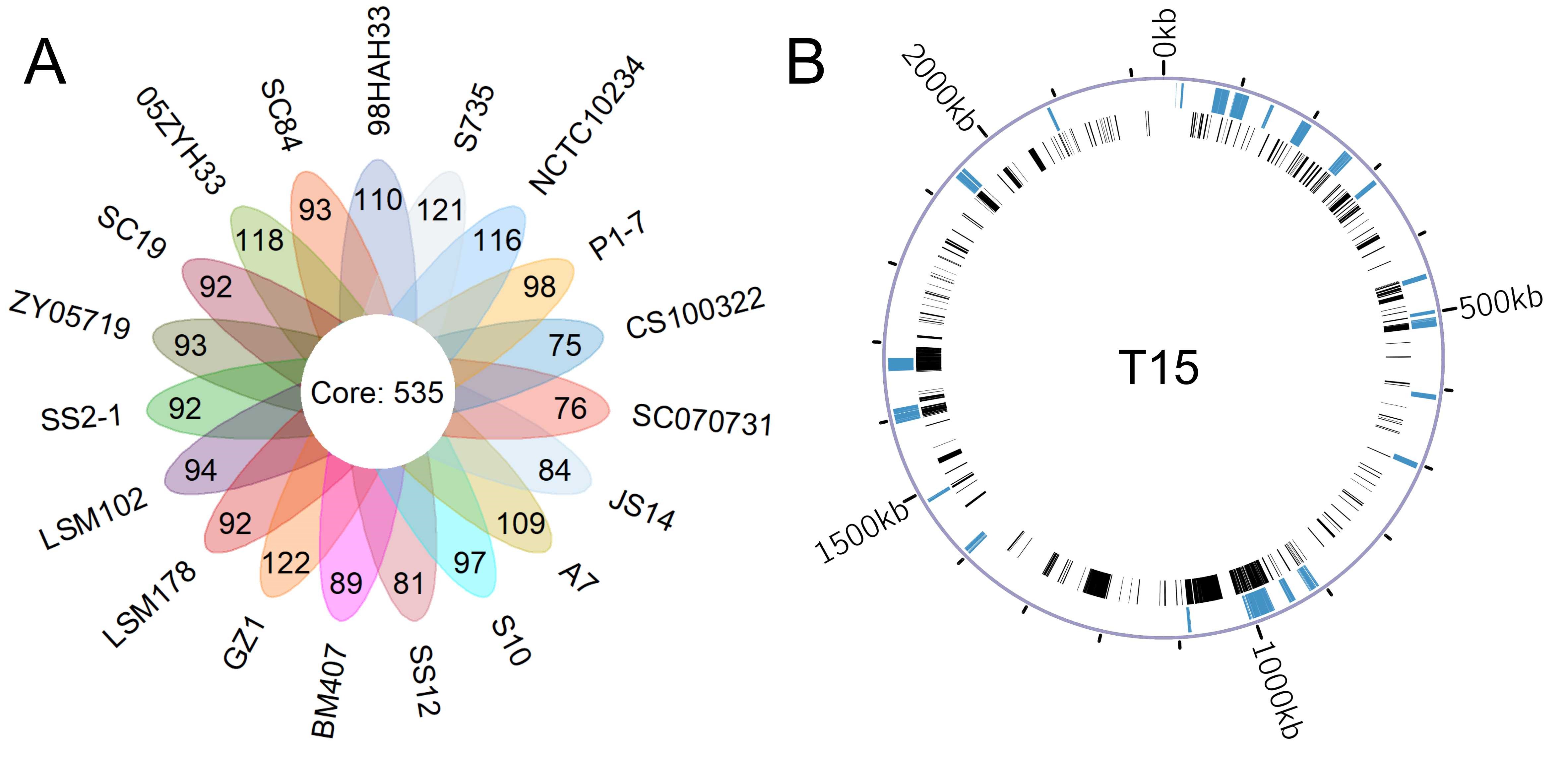

Supplement: Supplementary file 7 — Supplementary Information 7. [file 41598_2021_3370_MOESM7_ESM.jpg]
