# Supplementary material for: Genome analysis provides insight into hyper-virulence of Streptococcus suis LSM178, a human strain with a novel sequence type 1005
Source: Sci Rep. 2021 Dec 14;11:23919. doi: 10.1038/s41598-021-03370-0 (PMC8671398; doi:10.1038/s41598-021-03370-0)
Supplement: Supplementary file 8 — Supplementary Information 8. [file 41598_2021_3370_MOESM8_ESM.docx]

**Supplementary Tables.**

Table 1. Toxin-antitoxin (TA) components detected in LSM178 genome.

Table 2. Virulence factors found in LSM178 and other 52 *S. suis*.

Table 3. Details of the 52 *S. suis* and LSM178.

**Supplementary Figure 1.** Survival rate of zebrafish infected with LSM178, SC19 and P1/7 in two additional independent determinations. Zebrafish injected with PBS were used as the control. Each group contained 10 zebrafish.

**Supplementary Figure 2**. Dendrogram of *S. suis* isolates. All deposited STs were used in complexes analysis. Main clonal complexes including all STs from 52 *S. suis*  were illustrated. ST1005 was shown with red. The image was generated by goeBURST v1.2.1.

**Supplementary Figure 3**. Comparison of the biofilm formation ability of LSM178, SC19 and P1/7. P < 0.05 ­*, P < 0.01 ­**.

**Supplementary Figure 4**. Alignment of the PBPs of LSM178 with those of A7 and R61. A-E represented PBP1b (00760), PBP1a (02025), PBP1a (09665), PBP2b (03000) and PBP2x (08260) respectively.

**Supplementary Figure 5**. Alignment of GyrA and ParC from the LSM178 with those from quinolone-sensitive strains (A7, BM407, P1/7 and SC84).

**Supplementary Figure 6**. Alignment of ANT(6)-Ia from LSM178 with that from *Staphylococcus aureus* (UniProtKB - P12055 (AADK_STAAU)).

**Supplementary Figure 7**. (A) Venn diagram of the extracted genes of T15 in relative to each strain from LSM178 clade. The overlap was named core avirulence genes. (B) The location of GIs (blue) and core avirulence genes (black) in T15 genome. The images of A was generated using RStudio v4.0.5. The image of B was generated using circos v0.69.
